# Phenotypic Variations in a Large Family with Dominant Optic Atrophy Related to a Novel *OPA1* Deletion

**DOI:** 10.1016/j.xops.2026.101286

**Published:** 2026-06-15

**Authors:** Aymane Bouzidi, Patrizia Amati-Bonneau, Valérie Desquiret-Dumas, Cléis Beaulieu, Agnès Guichet, Céline Bris, Béatrice Bocquet, Xavier Dieu, Pascal Reynier, Delphine Mirebeau-Prunier, Clara Houdayer, Marc Planes, Sylvie Odent, Isabelle Meunier, Dominique Bonneau, Majida Charif, Guy Lenaers, Xavier Zanlonghi

**Affiliations:** 1 University of Angers, MitoLab, MitoVasc Unit, UMR CNRS 6015, INSERM U1083, SFR ICAT, University Hospital of Angers, Angers, France; 2 Department of Biochemistry and Molecular Biology, University Hospital of Angers, Angers, France; 3 Association Ouvrir Les Yeux, Lens, France; 4 Department of Genetics, University Hospital of Angers, Angers, France; 5 Institute for Neurosciences of Montpellier, University of Montpellier, INSERM, Montpellier, France; 6 National Reference Center for Inherited Sensory Diseases, University of Montpellier, CHU, Montpellier, France; 7 Department of Pediatrics and Medical Genetics, Brest University Hospital, Brest, France; 8 Clinical Genetics, CLAD-West Rare Disease Reference Center, ERN-ITHACA, GenOMedS FHU, Rennes University Hospital, Rennes, France; 9 Department of Neurology, University Hospital of Angers, Angers, France; 10 Department of Genetics, University Hospital of Orleans, Orleans, France; 11 Genetics and Immuno-Cell Therapy Team, Faculty of Sciences, Mohammed First University, Oujda, Morocco; 12 Department of Ophthalmology, Rare Disease Competence Center, Rennes University Hospital, Rennes, France

**Keywords:** Dominant optic atrophy, OPA1, Phenotypic variation, Visual acuity evolution

## Abstract

**Purpose:**

Dominant optic atrophy (DOA) is a rare disease characterized by the chronic loss of retinal ganglion cells that transduce the visual information from the retina to the brain. Dominant optic atrophy shows interfamilial and intrafamilial phenotypic variations and a restricted 35% molecular diagnosis, with half presenting a pathogenic variant in optic atrophy 1 (*OPA1*), encoding a large mitochondrial GTPase. Here, we describe the largest ever identified DOA family, with 64 nonsyndromic individuals harboring a novel 10-kb *OPA1* deletion.

**Design:**

Retrospective, longitudinal cohort study of a family from the Western part of France.

**Participants:**

Thirty-nine individuals were included, 34 patients harboring a novel *OPA1* 10-kb deletion and 5 healthy controls.

**Methods:**

*OPA1* sequencing, multiplex ligation-dependent probe amplification, and copy number variations using single nucleotide polymorphism array identified the genetic variation causing DOA. The relatedness between the different family branches was analyzed by micro-satellite markers. Best-corrected visual acuity (BCVA), Lanthony D-15 desaturated color vision test, retinal nerve fiber layer (RNFL), and macular ganglion cell layer (GCL) thickness were recorded at 2 different time points for longitudinal analyses.

**Main Outcome Measures:**

Best-corrected visual acuity, RNFL and GCL thickness at first and follow-up examinations. Correlations between morphological and functional measurements.

**Results:**

We disclosed the largest ever identified DOA family, with 64 nonsyndromic patients for whom we discovered a novel 10-kb deletion encompassing *OPA1* exons 30 and 31. Ophthalmic examination revealed a consistent BCVAvariability, ranging from 0 (Snellen equivalent, 20/20) to 1.61 (20/815) logarithm of the minimum angle of resolution (logMAR), strongly correlated to RNFL and GCL thickness, but moderately with age and not with dyschromatopsia. Follow-up of individuals evidenced a significant BCVA loss with a median of 0.018 logMAR per year (0.18 logMAR per decade) and a temporal, superior, and inferior RNFL thickness loss of 1.13, 0.70, and 0.50 μm/yr, respectively, whereas the nasal quadrant did not evolve.

**Conclusion:**

The identification of a large *OPA1* deletion in this DOA family illustrates the critical importance of screening for large genomic rearrangements in DOA genes and confirms the high intrafamilial phenotypic variability while correlating BCVA with RNFL and GCL thickness.

**Financial Disclosure(s):**

The authors have no proprietary or commercial interest in any materials discussed in this article.

Leber and Kjer hereditary optic neuropathies (HONs) form heterogeneous groups of genetic diseases associated with acute and chronic visual loss, respectively. They are caused by the selective dysfunction followed by degeneration of the retinal ganglion cells, whose axons constitute the optic nerve and transduce the visual information from the retina to the brain.^1^ Both diseases are related to mitochondrial dysfunctions, Leber HON being typically associated with 3 mitochondrial genome variants,^2^, ^3^, ^4^ whereas Kjer HON, known as dominant optic atrophy (DOA), is associated with nuclear gene variants, mainly in the optic atrophy 1 (*OPA1*) gene.^5^, ^6^, ^7^

In DOA cases, the onset of visual decline usually occurs in the first or second decade of life, although identifying a precise onset timing is challenging due to the gradual decrease in the visual acuity.^1^^,^^8^^,^^9^ Dominant optic atrophy visual loss is typically bilateral and symmetrical, but highly variable between families sharing the same molecular defect, or inside a family, ranging from pauci-symptomatic (<0.2 logarithm of the minimum angle of resolution [logMAR]; 20/32) to legal blindness (>1.0 logMAR; 20/200).^10^ However, most often, patients present with moderate loss of visual acuity in the range of 0.2 (20/32) to 0.6 logMAR (20/80), with preserved peripheral visual fields, disclosing a restricted central or cecocentral scotoma.^11^^,^^12^ In addition, many DOA cases present a pathognomonic blue–yellow dyschromatopsia, although absence of, or mixed color deficit, is common.^12^ Fundus examination reveals in most cases a pallor of the optic nerve head in both eyes, predominantly in the temporal quadrant in the fewer affected ones while being more diffuse in the most severely affected individuals.^13^ Retinal nerve fiber layers (RNFLs) and macular ganglion cell layer (GCL) assessments by OCT imaging confirm the primary thinning of the papillomacular bundle,^13^, ^14^, ^15^ corresponding to the central visual field defect.

Dominant optic atrophy molecular diagnosis is mainly associated with variants in 3 genes: *OPA1*, Wolfram syndrome 1, and aconitase 2, *OPA1* being responsible for >50% of all cases.^5^^,^^16^^,^^17^ *OPA1* variants resulting in haploinsufficiency are in general responsible for a nonsyndromic presentation, whereas dominant-negative variants can induce syndromic presentations, named DOAplus, including primarily a neurosensorial deafness, and eventually a peripheral neuropathy and/or a myopathy.^12^^,^^18^ Additional causative genes were identified in recent years; nevertheless, their systematic screening by panel or exome sequencing only provides a successful result in 1 out of 3 cases,^5^^,^^16^^,^^17^ suggesting that alternative molecular features such as deep intronic mutations, large deletions, or chromosomal rearrangements might be responsible for this disease, as illustrated in few *OPA1* cases.^19^, ^20^, ^21^, ^22^, ^23^

Here, we describe the largest DOA family ever reported, in which we identified a 10-kb deletion encompassing *OPA1* exons 30 and 31. Extensive ophthalmic evaluation and follow-up of 34 individuals out of the 64 affected individuals illustrate a high variability of the visual alteration, but tight correlations between best-corrected visual acuity (BCVA), RNFL, and GCL measurements, whereas follow-up data suggest that the disease evolves faster before 30 years of age than later and in women compared with men.

## Methods

### Participants

**Figure 1:**
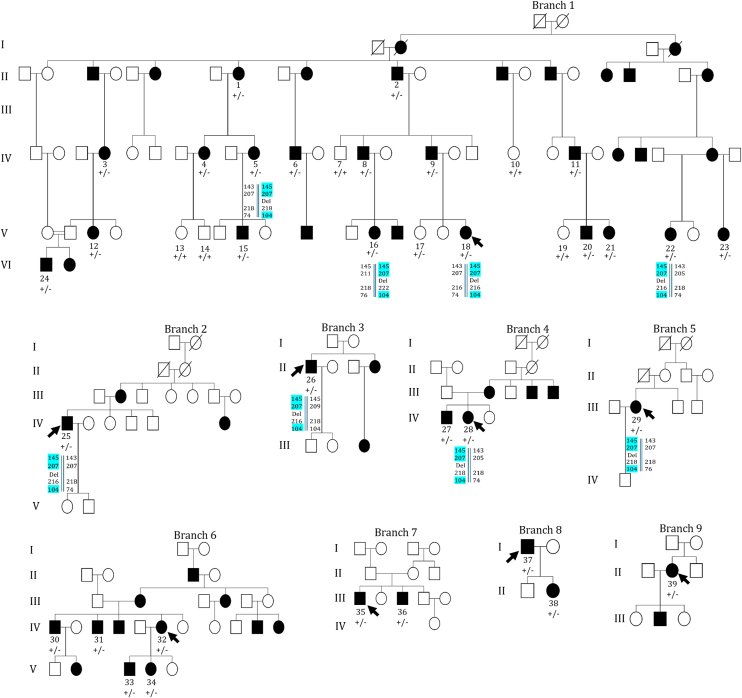
Pedigrees of the 9 branches of the family with the optic atrophy 1 exons 30 and 31 deletion. Numbers 1 to 39 indicate individuals with a molecular diagnosis and a clinical examination (see Table S1). Clinically affected individuals are presented with filled symbol and unaffected with open symbol. Wild-type homozygotes and optic atrophy 1-mutated heterozygotes are represented with +/+ and +/–, respectively. The arrows indicate the index patient for each branch. Microsatellite lengths for each marker (top to bottom: D3S3642, D3S3590, D3S3562, and D3S2748) are presented below each branch representative and colored in blue when identical between branches.

We report a cohort study of a single family comprising 9 branches and 64 individuals affected with DOA. Thirty-nine individuals were examined, among which 34 displayed DOA and a 10-kb deletion encompassing *OPA1* exons 30 and 31, whereas 5 were unaffected wild-type controls (Fig 1). All procedures were in accordance with the tenets of the Declaration of Helsinki. Institutional review board approval was obtained from the University Hospitals of Angers and Rennes, and informed consent was collected and signed by all individuals after explanation of the nature and possible consequences of the study. Informed consent was further obtained to publish the clinical information and images.

### Clinical Examination

Collected data included age of onset, sex, BCVA with Snellen charts (converted to the logMAR using the formula logMAR=–log10(Snellenfraction), ocular fundus imaging (Nidek nonmydriatic automated fundus camera, AFC 330, Nidek Inc), Lanthony D-15 desaturated color vision test, retinal nerve fiber, and macular analyses, using the spectral domain OCT (Combined Heidelberg Retina Angiograph, Spectralis OCT Device, Heidelberg Engineering). Retinal nerve fiber layer thickness in micrometers was extracted for the global and sectoral regions using spectral domain OCT with a 12° circular scan centered on the optic disc. Macular GCL sectoral thickness values were obtained from the 3-mm ETDRS grid (inner ring) centered on the fovea.

Best-corrected visual acuity and RNFL data were collected at 2 distinct time points for 26 and 13 individuals, respectively, and reported as BCVA1 or BCVA2, and RNFL1 or RNFL2, respectively (Table S1, available at www.ophthalmologyscience.org). All individuals were examined by a single medical doctor and answered a quality of life and an environmental questionnaire to identify confounder parameters.

### Genetic Analyses

Total DNA was extracted from peripheral blood with a NucleoMag Kit (Macherey-Nagel), using a Starlet Presto (Hamilton, Thermo Fisher Scientific) automated sample process.

The index patient of branch 1 (individual 18) was screened first for *OPA1* pathogenic variants in all coding exons using direct Sanger sequencing because clinical evidence supported *OPA1*-related DOA. Because no pathogenic variant was found after the first screening, targeted next-generation sequencing using a panel of 87 nuclear genes was applied as described,^5^ without success.

Next, multiplex ligation-dependent probe amplification (MLPA) targeting all *OPA1* exons using the Selective Adaptor Ligation, Selective Amplification MLPA Probemix P229 OPA1 (MRC Holland) was performed following the manufacturer’s instructions, and fragments were separated on a 3500xL Genetic Analyzer (Thermofisher Scientific). Data analysis using the Coffalyser.Net, version 220513.1739 (MRC Holland), disclosed a 50% reduction of probes targeting *OPA1* exons 30 and 31.

Copy number variation (CNV) analysis using the Illumina single nucleotide polymorphism-array Infinium CytoSNP-850K v1.2 BeadChip (Illumina) and the Illumina NextSeq550/iScan system was performed following the manufacturer’s instructions. Data analyses were conducted with Genome Studio v2.0.4 (Genotyping module and Illumina Genome Viewer 2.0.4) and CNV partition v3.2.0, the Cartagenia BENCHLab CNV v5.1.9 (Agilent Technologies).

To determine the genomic coordinates of the deletion, next-generation sequencing was used to sequence the full *OPA1* gene. In brief, targeted enrichment of *OPA1* was performed using Agilent SureSelect Capture custom DNA kit (Agilent Technologies, Inc). Custom capture probes were designed with the Agilent SureDesign website (https://earray.chem.agilent.com/suredesign/) based on the GRCh38 reference genome. Library preparation, including hybridization and capture, was performed according to the manufacturer’s instructions (manual number G7530-90005). Emulsion polymerase chain reaction and enrichment were performed using pooled libraries and an Ion Chef and sequenced on the Ion S5-XL (Thermo Fisher Scientific), according to the manufacturer’s protocol.

**Figure 2:**
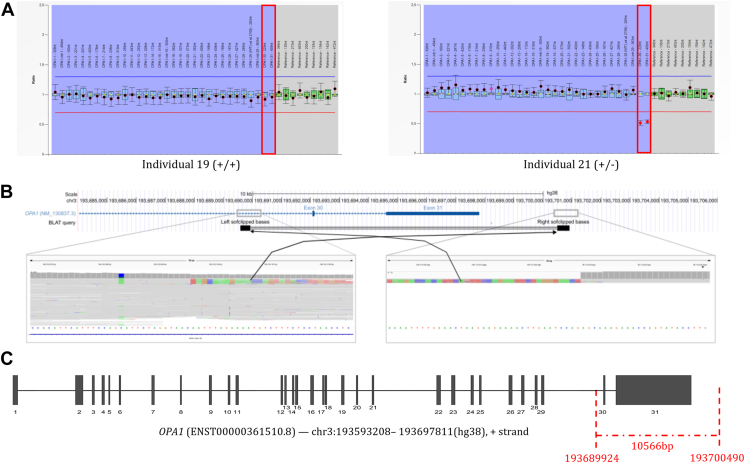
Characterization of the optic atrophy 1 (*OPA1*) ex30-31 deletion. **A,** Inter-ratio profile of the multiplex ligation-dependent probe amplification probemix P229 *OPA1* of a wild type (WT) individual (#19, left) and a dominant optic atrophy individual (#21, right), illustrating the 50% decrease of the signal corresponding to exons 30 and 31. The blue portion corresponds to the 32 *OPA1* probes, and gray 1 represents the quality control fragments. The black dots represent the probes that are expressed in a normal ratio (0.8–1.2), and the red dots represent the probes that are underexpressed (ratio under 0.7). **B,** Visualization of soft-clipped sequences by Integrative Genomics Viewer and their genomic coordinates, as shown with the University of California, Santa Cruz Genome Browser Interface. **C,** Schematic representation of *OPA1* exons. Dashed red lines mark the genomic positions of the deletion limits.

Based on MLPA information and sequencing coverage data, we inspected the alignments of reads in this region from binary alignment map files, using Integrative Genomics Viewer (V.2.19.1) (Fig 2B). The exact deletion breakpoints (Fig 2B) were determined by visualization of soft-clipped sequences upstream of exon 30 and downstream of exon 31, followed by a local alignment using the Blast software and a visualization in the University of California, Santa Cruz Genome Browser Interface.^24^

In addition, microsatellite markers (D3S3642, D3S3590, D3S3562, and D3S2748) were analyzed to assess the conservation of surrounding sequences and to explore the relatedness between the family branches. Polymerase chain reaction primer sequences were obtained from GeneLoc (https://geneloc.weizmann.ac.il/index.shtml), in which the forward primers were 5’-labeled with 6-carboxyfluorescein. Polymerase chain reaction products were mixed with the GeneScan 600-LIZ fluorescent size Standard v2.0 (Applied Biosystems) and analyzed by capillary electrophoresis on an ABI PRISM 3500xL Genetic Analyzer (Applied Biosystems). Fragment lengths were analyzed with Gene Mapper Software (Applied Biosystems).

### Statistical Analyses

Medians and interquartile ranges were calculated for all quantitative variables. The Shapiro–Wilk test was used to evaluate the normal distribution of the data. Wilcoxon rank-sum test (i.e., Mann–Whitney *U* test) was used for groups’ comparison. Exact *P* values were computed when possible; otherwise, a normal approximation with continuity correction was applied as implemented in R.

Pearson’s (r) and Spearman’s (ρ) correlations were used to assess the linear associations between age, BCVA, RNFL, and GCL. Pearson’s correlations were calculated for normally distributed variables and are presented as r (95% confidence interval), whereas Spearman’s correlations were used for non-normally distributed variables and presented as ρ. A *P* value of <0.05 is considered statistically significant. To obtain reliable correlation analyses, measurements performed at equivalent ages were prioritized. To test if BCVA and RNFL evolution per year is different from 0, the one-sample Wilcoxon signed-rank test was applied. Statistical analyses were performed using R version 4.4.1, using the gtsummary (2.3.0)^25^ and tidyverse (2.0.0)^26^ packages (The R Foundation for Statistical Computing, available at: http://www.r-project.org).

## Results

### Molecular Findings

We describe a novel *OPA1* deletion encompassing 10.566 bp (Fig 2C) in 34 DOA individuals belonging to 9 branches of a single family (Fig 1).

This deletion was first discovered by MLPA analysis, which evaluates the CNVs of all *OPA1* exons. The signal ratio, corresponding to the DNA amount detected for both alleles compared with a reference control, showed a 50% reduction of the signal for the coding exon 30 and the noncoding exon 31 in DOA individuals (Fig 2A). Healthy relatives from the same branches showed normal ratios for all exons. This result was further supported by a CNV analysis that indicated a loss of heterozygosity of 5 single nucleotide polymorphisms in the region covering exons 30 and 31. The deletion breakpoints and their genomic coordinates were obtained by Blast of soft-clipped reads from *OPA1* next-generation sequencing data in this region (Fig 2A, B). Consequently, MLPA was performed on all family members.

To explore the relatedness among branches and the possibility of a founder effect, short tandem repeat analyses were performed and revealed a conservation of 3 short tandem repeat markers (D3S3642, D3S3590, and D3S2748) around the deleted region (Fig 1), illustrating a common haplotype witnessing the relatedness between all individuals assessed and a common ancestor.

### Clinical Findings

**Figure 3:**
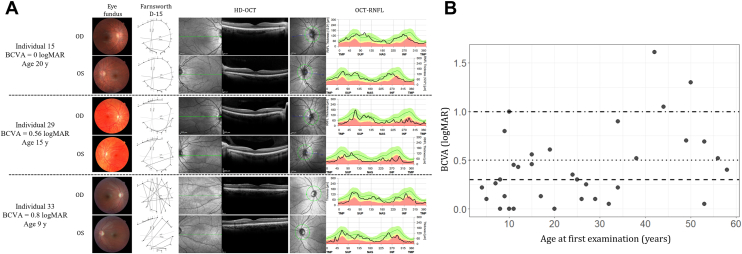
Clinical heterogeneity in dominant optic atrophy individuals with optic atrophy 1 ex30-31 deletion. **A,** Phenotypic variations of the BCVA, eye fundus, color vision, and RNFL of 3 individuals with the optic atrophy 1 ex30-31 deletion, from the less affected (top) to the most affected (bottom). **B,** Scatter plot displaying the relationship between BCVA and age at first examination. Dashed lines indicate visual impairment categories as defined by the International Classification of Diseases 11 classification; 0 to 0.3 logMAR (20/20–20/40): no visual impairment, 0.3 to 0.5 logMAR (20/40–20/63): mild visual impairment, 0.5 to 1 logMAR (20/63–20/200): moderate visual impairment, and >1 logMAR (20/200): severe visual impairment. BCVA = best-corrected visual acuity; HD-OCT = high-definition OCT; INF = inferior; logMAR = logarithm of the minimum angle of resolution; NAS = nasal; OD = oculus dexter; OS = oculus sinister; RNFL = retinal nerve fiber layer; SUP = superior; TMP = temporal.

Overall, individuals with *OPA1* ex30-31 deletion presented with decreased BCVA, pallor of the optic nerve head predominantly on the temporal side, tritan dyschromatopsia, and RNFL thinning (Fig 3A), however with a high variability between and within branches. None of the individuals displayed relevant confounding parameters that could significantly affect the ophthalmic examination and results. Best-corrected visual acuity ranged from 0 (20/20; asymptomatic) to 1.61 (20/815; severe vision impairment) logMAR at first examination, with a median of 0.33 logMAR (20/43) (Fig 3 and Tables 1 and S1). Based on the International Classification of Diseases 11^27^ visual impairment thresholds, 15 out of 34 individuals with the *OPA1* deletion had no visual impairment (BCVA < 0.3 logMAR; 20/40), 7 out of 34 had a mild visual impairment (0.3 logMAR; 20/40 ≤ BCVA < 0.5 logMAR; 20/63), 8 out of 34 had moderate visual impairment (0.5 logMAR; 20/63 ≤ BCVA < 1 logMAR; 20/200), and only 4 out of 34 had a severe visual impairment (BCVA ≥ 1 logMAR; 20/200) (Fig 3B).

**Table 1:** Clinical Characteristics of Patients with the Optic Atrophy 1 ex30-31 Deletion and Their Controls (Unaffected Relatives)

		Control∗		Patient∗		*P* Value†^,^‡
BCVA (logMAR) Snellen equivalent		n = 5	0.00 (0.00–0.00) 20/20	n = 34	0.33 (0.10–0.61) 20/43 (20/25–20/81)	**0.003**
RNFL	Global (μm)	n = 3	103 (78–109)	n = 31	67 (57–80)	**0.03**
	Temporal (μm)		63 (60–77)		33 (26–44)	**0.01**
	Superior (μm)		127 (92–145)		97 (85–109)	0.14
	Nasal (μm)		82 (56–86)		57 (51–71)	0.20
	Inferior (μm)		132 (103–137)		79 (63–94)	**0.03**
GCL	Average thickness (μm)		NA	n = 18	22 (19–31)	
	Temporal (μm)		NA		21 (14–28)	
	Nasal (μm)		NA		19 (14–25)	
	Superior (μm)		NA		26 (17–36)	
	Inferior (μm)		NA		23 (15–29)	

BCVA = best-corrected visual acuity; GCL = ganglion cell layer; logMAR = logarithm of the minimum angle of resolution + Snellen equivalent; NA = not available; RNFL = retinal nerve fiber layer.

∗ Median (Q1–Q3).

† Wilcoxon rank-sum test with continuity correction.

‡ Bold values highlight significant values.

In addition, patients presented with various degrees of color vision defect, with 35.3% displaying normal color perception (n = 12), 44.1% exhibiting moderate/severe tritan defect (n = 15), and 11.8% displaying anarchic color vision (n = 4) (Fig 3A and Table S1).

Comparison of RNFL quadrants’ thickness between patients with DOA (n = 31) and controls (n = 3) disclosed a significant thinning of the temporal (47.6% decrease, *P* = 0.01) and inferior (40.1% decrease, *P* = 0.03) regions (Table 1). Thinning in the superior (23.6%, *P* = 0.14) and nasal (30.5%, *P* = 0.20) quadrants was not statistically significant.

Next, we investigated the correlations between BCVA, RNFL, GCL, and age. All reported correlations are based on 2-tailed tests with a significance threshold set at α = 0.05 (Table 2).

**Table 2:** Correlations between Morphological and Functional Measurements in Patients with the Optic Atrophy 1 ex30-31 Deletion

		n	Age		BCVA (LogMAR)	
			Correlation∗	P Value†	Correlation∗	P Value†
BCVA (logMAR)		34	0.35	**0.04**		
RNFL	Global (μm)	31	–0.42	**0.02**	–0.74 (–0.87 to –0.52)	**<0.001**
	Temporal (μm)		–0.51	**0.003**	–0.63	**<0.001**
	Superior (μm)		–0.53	**0.002**	–0.72 (–0.85 to –0.49)	**<0.001**
	Nasal (μm)		0.08	0.65	–0.50	**0.004**
	Inferior (μm)		–0.45	**0.01**	–0.73 (–0.86 to –0.51)	**<0.001**
GCL	Average thickness (μm)	18	–0.55	**0.02**	–0.86	**<0.001**
	Temporal (μm)		–0.57	**0.01**	–0.82	**<0.001**
	Superior (μm)		–0.53 (–0.8 to –0.08)	**0.02**	–0.85 (–0.94 to –0.62)	**<0.001**
	Nasal (μm)		–0.59	**0.01**	–0.85	**<0.001**
	Inferior (μm)		–0.61	**0.008**	–0.86	**<0.001**

BCVA = best-corrected visual acuity; GCL = ganglion cell layer; logMAR = logarithm of the minimum angle of resolution + Snellen equivalent; RNFL = retinal nerve fiber layer.

∗ Pearson’s (r) and Spearman’s (ρ) correlation coefficients were calculated for normally and non-normally distributed data, respectively. Pearson’s correlations are presented as r (95% confidence interval).

† Bold values highlight significant values.

Moderate correlations were observed between age at first visit and BCVA (ρ = 0.35; n = 34; *P* = 0.04), and age and RNFL thickness (–0.53 ≤ ρ ≤ –0.45; n = 31; *P* < 0.05), except for the nasal quadrant (ρ = 0.08; *P* = 0.65). Ganglion cell layer thickness significantly correlated with age (–0.61 ≤ r/ρ ≤ –0.53; n = 18; *P* < 0.05) across all quadrants (Table 2).

Strong correlations were found between BCVA and RNFL thickness (–0.73 ≤ r/ρ ≤ –0.50; n = 31; *P* < 0.005) (Table 2) and between BCVA and GCL components (–0.86 ≤ r/ρ ≤ –0.82; *P* < 0.001; n = 18). Scatter plots illustrating these correlations (Figs S1–S4, available at www.ophthalmologyscience.org) revealed that GCL measurements accurately reflect disease progression with age and BCVA.

**Figure 4:**
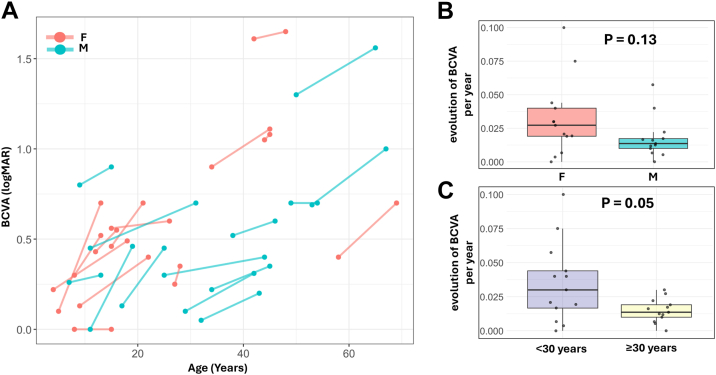
Evolution of the BCVA between 2 examinations. **A,** Scatter plot illustrating the evolution of BCVA between 2 clinical examinations. Line colors show gender differences. **B,** Boxplot showing the evolution difference of BCVA per year between males and females. **C,** Boxplot displaying the evolution difference of BCVA per year between dominant optic atrophy individuals before or after the age of 30 years. BCVA = best-corrected visual acuity; F = female; logMAR = logarithm of the minimum angle of resolution; M = male.

We further assessed the evolution of BCVA and RNFL thickness between 2 successive clinical examinations (Fig 4) for 26 and 13 individuals, respectively. A yearly decline rate was estimated, and a 1-sample Wilcoxon signed-rank test (2-tailed, α = 0.05) was used to assess whether the decline rate by year differed significantly from 0 (Table 3).

**Table 3:** Evolution of the Visual Acuity and Retinal Nerve Fiber Layer between 2 Clinical Examinations in Patients with Optic Atrophy 1 ex30-31 Deletion

		n	First Examination		Second Examination		Years of Follow-Up∗	Decline Rate per Year∗	*P* Value†^,^‡
			Age∗	Value∗	Age∗	Value∗			
BCVA (logMAR) Snellen equivalent		26	21 (9–38)	0.35 (0.13–0.69) 20/45 (20/27–20/98)	30 (18–45)	0.58 (0.40–0.70) 20/76 (20/50–20/100)	8.0 (6.0–13.0)	0.018 (0.010–0.030)	**<0.001**
RNFL	Global (μm)	13	14 (7–31)	73 (65–84)	20 (16–40)	67 (60–74)	7.16 (6.33–8.28)	–0.75 (–1.00 to –0.40)	**0.002**
	Temporal (μm)			34 (31–44)		28 (25–36)		–1.13 (–1.44 to –0.33)	**0.002**
	Superior (μm)			101 (94–118)		100 (90–104)		–0.70 (–1.42 to –0.20)	**0.001**
	Nasal (μm)			61 (52–78)		60 (48–73)		–0.50 (–0.78 to 0.17)	0.16
	Inferior (μm)			84 (76–107)		76 (64–102)		–0.50 (–2.06 to 0.06)	**0.048**

BCVA = best-corrected visual acuity; logMAR = logarithm of the minimum angle of resolution + Snellen equivalent; RNFL = retinal nerve fiber layer.

∗ Median (Q1–Q3).

† One-sample Wilcoxon signed-rank test (2-tailed) with continuity correction.

‡ Bold values highlight significant values.

We observed a median gain of 0.018 logMAR per year in *OPA1* ex30-31del patients, which corresponds to the loss of 1 letter on the logMAR chart per year, and almost 2 lines per decade. This decline rate is different from 0 (n = 26; V = 300; *P* < 0.001; 95% confidence interval, 0.015–0.03) (Table 3). Sex-specific analysis revealed that females (n = 13) display a higher yearly decline rate than males (n = 13), with a median of 0.027 and 0.014 logMAR, although the difference between both groups was not significant (W = 53.5; *P* = 0.13; 95% confidence interval, –0.024 to 0.005) (Fig 4B). Individuals examined before the age of 30 presented a higher BCVA decline rate (0.030 logMAR/yr) than those examined after the age of 30 (0.014 logMAR/yr), without a significant difference between both groups (n = 13; W = 122.5; *P* = 0.05) (Fig 4C). Temporal and superior RNFL quadrants presented the highest decline rate, with a loss of 1.13 μm (*P* < 0.002) and 0.70 μm (*P* < 0.001) per year, respectively, whereas a decline rate of 0.50 μm/yr was observed in the inferior (*P* = 0.048) and nasal (*P* = 0.16) quadrants (Table 3).

## Discussion

In this study, we describe the largest DOA family ever reported, including 64 affected individuals. Among them, 34 had a genetic analysis that disclosed a novel 10-kb deletion of *OPA1*, encompassing exons 30 and 31 and downstream sequence. Due to incomplete historical data, it was not possible to establish definitive links between the different branches to draw a complete, unified pedigree. However, all affected individuals belonged to a French Manouche community settled in the Western part of France and presented the same *OPA1* deletion and 3 conserved short tandem repeat markers around the deletion, supporting a founder family effect with a unique common ancestor.

Heterozygous *OPA1* deletions of various lengths have been reported, ranging from few base pairs^19^ to one or more exons,^20^, ^21^, ^22^, ^23^^,^^28^ or to the entire gene,^20^^,^^22^ the latter supporting the evidence that *OPA1* haploinsufficiency is one cause of DOA. Other studies reported 3q28 to 29 deletions ranging from 560 to 1630 kb, encompassing *OPA1* and adjacent genes, and associated with syndromic DOA. Extraocular features included *pes cavus*, reduced deep tendon reflexes and toe-walking,^29^ brain aneurysm,^30^ intellectual disability, psychiatric disorders, and obesity^31^ that may or may not be related to *OPA1* haploinsufficiency. Additionally, among patients diagnosed with Behr syndrome, a recessive *OPA1* presentation caused by the co-occurrence of a loss-of-function variant in trans with a hypomorphic variant,^32^ one harbored a *de novo* 3975-kb deletion combined with a heterozygous missense variant (*OPA1*:NM_015560: c.2189 T > C p.Leu730Ser) inherited from his unaffected father.^33^ Nevertheless, the heterozygous *OPA1* deletion identified here, which does not affect the coding sequence of another gene, is associated with a pure nonsyndromic DOA.

Recently, we provided an overview of HON molecular diagnosis from 2186 patients recruited in the last 5 years,^5^ in which 166 individuals displayed an *OPA1* variant, accounting for 37% of all autosomal cases. Combining the previous cohort with the 34 affected individuals from this study improves the *OPA1* molecular diagnosis rate by 17% (34/200) and should prompt systematic investigation of *OPA1* exon CNV to improve HON diagnostic rate and overcome limitations of short-read sequencing technologies.

Dominant optic atrophy is characterized by a progressive optic nerve degeneration typically starting in early childhood, and manifesting as heterogeneous bilateral vision loss and color vision defects with a high variability of clinical severity.^34^ However, the primary diagnosis often occurs later, during early adulthood, when the visual defect becomes prominent.^34^ This is exemplified in our cohort, with a median age at first examination of 22 years of age (IQR, 10–38 years) and a median BCVA of 0.33 logMAR (20/43; IQR, 0.10–0.61), illustrating the high clinical variability with a moderate correlation between these 2 parameters (ρ = 0.35; *P =* 0.04). Follow-up data on a median of 8.0 years (IQR, 6.0–13.0 years) disclosed that 24 out of 26 individuals (92.3% of our cohort) experienced vision changes, with median and average progression rates of 0.018 and 0.025 logMAR/year, respectively. These data differ from a previous cohort study of 43 individuals encompassing haploinsufficient and dominant-negative *OPA1* variants, with a mean follow-up duration of 18.0 years (range, 1.2–53.0 years),^10^ in which only 67.4% of patients displayed BCVA reduction over time, but with a higher mean vision loss rate of 0.032 logMAR/year. This difference might be due to the nature of *OPA1* variants because our study only includes patients with a single deletion variant, predicted to exert a milder pathological effect than *OPA1* missense variants.^10^

Our study suggests a tendency toward accelerated vision loss in women (0.027 logMAR/yr, n = 13) compared with men (0.014 logMAR/yr, n = 13), which we already reported in a former study based on a larger DOA cohort (n = 154) with different types of *OPA1* variants.^35^ Similarly, we found a tendency toward an accelerated BCVA decline in patients aged <30 years (0.030 logMAR/yr, n = 13), compared with older patients (0.014 logMAR/yr, n = 13).

Comparison of sectoral thickness mapping using spectral domain OCT between DOA individuals and controls revealed that the temporal and inferior RNFL quadrants are the most affected, with a median reduction of 47.6% and 40.1%, respectively. Several studies showed similar results,^13^, ^14^, ^15^^,^^36^ highlighting that RNFL thickness is reduced in the temporal (–47.0% to –52.4%) and the inferior (–40% to –45.8%) quadrants in patients with DOA, compared with controls.^15^^,^^37^ In these studies, linear regression was used to infer age-related RNFL thinning, revealing that the yearly decline in RNFL thickness is significantly more pronounced in the superior and inferior quadrants^14^^,^^15^ and milder for the nasal and temporal quadrants. However, our longitudinal data disclosed a higher decline rate in the temporal (1.13 μm/yr) and superior (0.70 μm/yr) quadrants (Table 3).

In addition, GCL measurements displayed stronger correlations with age and with BCVA than RNFL did, in line with previous studies showing that retinal ganglion cell loss in the macula precedes peripapillary RNFL atrophy and accurately reflects DOA progression.^13^^,^^14^

Altogether, this study reports a novel *OPA1* deletion in the largest ever described DOA family, in which we evidenced strong correlations between BCVA, RNFL, and GCL thickness, despite a high intrafamilial clinical variability. We further disclosed a tendency toward accelerated vision loss in women and individuals aged <30 years. These observations should prompt systematic evaluation of gene rearrangements to improve the molecular diagnosis of HON and are critical to define future cohorts of patients for clinical trials and the best criteria to evaluate the DOA evolution among *OPA1* individuals.
