# Supplementary material for: Phenotypic Variations in a Large Family with Dominant Optic Atrophy Related to a Novel OPA1 Deletion
Source: Ophthalmol Sci. 2026 Jun 15;6(8):101286. doi: 10.1016/j.xops.2026.101286 (PMC13382307; doi:10.1016/j.xops.2026.101286)
Supplement: Supplementary Table 1 [file mmc1.pdf]

Supplementary table 1 : clinical data from family branch members with the *OPA1* ex30-31 deletion (DOA) and their healthy relatives (Control).

ID	Branch	Gender	Age BCVA1	BCVA1	Age BCVA2	BCVA2	Color vision		RNFL1 (µm)						RNFL2 (µm)						GCL (µm)						Phenotype
							OD	OS	Age	Global	Temporal	Superior	Nasal	Inferior	Age	Global	Temporal	Superior	Nasal	Inferior	Age	Average thickness	Temporal	Superior	Nasal	Inferior	
1	Branch 1	F	58	0.40	69	0.70	B/Y Moderate	B/Y Moderate	68	58	28.5	80	62	63	NA	NA	NA	NA	NA	NA	68	18.75	12	17	12	14	DOA
2	Branch 1	M	50	1.30	65	1.56	Anarchic	Anarchic	55	51	19.5	79	52	54.5	NA	NA	NA	NA	NA	NA	NA	NA	NA	NA	NA	NA	DOA
3	Branch 1	F	53	0.05	NA	NA	Normal	Normal	51	91	61.5	108.5	81.5	112.5	NA	NA	NA	NA	NA	NA	51	31.13	28	36	27.5	33	DOA
4	Branch 1	F	34	0.90	45	1.11	B/Y Moderate	B/Y Moderate	45	50	30.5	72	45	51.5	NA	NA	NA	NA	NA	NA	45	12.50	12	14	12	12	DOA
5	Branch 1	F	44	1.05	45	1.08	B/Y Moderate	B/Y Moderate	44	57	21.5	89	61.5	55.5	NA	NA	NA	NA	NA	NA	44	14.88	14	17	13.5	15	DOA
6	Branch 1	M	25	0.30	44	0.40	B/Y Moderate	B/Y Moderate	44	91.5	40	94.5	141	91.5	NA	NA	NA	NA	NA	NA	NA	NA	NA	NA	NA	NA	DOA
7	Branch 1	M	32	0.00	NA	NA	Normal	Normal	32	103	62.5	126.5	85.5	137	NA	NA	NA	NA	NA	NA	NA	NA	NA	NA	NA	NA	Control
8	Branch 1	M	29	0.10	42	0.31	B/Y Moderate	B/Y Moderate	31	66.5	30.5	101	55.5	79	40	64.5	27.5	100	51.5	81	40	20.63	21.5	25	17	19	DOA
9	Branch 1	M	34	0.22	45	0.35	B/Y Moderate	B/Y Moderate	34	74.5	30.5	101	73.5	92.5	44	70.5	28.5	102	65	85.5	44	21.13	19.5	24.5	19	21.5	DOA
10	Branch 1	F	29	0.05	NA	NA	Normal	Normal	29	78	60	91.5	55.5	102.5	NA	NA	NA	NA	NA	NA	NA	NA	NA	NA	NA	NA	Control
11	Branch 1	M	32	0.05	43	0.20	B/Y Moderate	B/Y Moderate	32	68.5	25	88.5	85	75.5	41	66.5	22	89	78	76	41	20.25	18.5	27.5	17	18	DOA
12	Branch 1	F	26	0.10	NA	NA	B/Y Moderate	Normal	26	72	34	119	56.5	78	NA	NA	NA	NA	NA	NA	26	24.75	20	31.5	23.5	24	DOA
13	Branch 1	F	7	0.00	NA	NA	Normal	Normal	NA	NA	NA	NA	NA	NA	NA	NA	NA	NA	NA	NA	NA	NA	NA	NA	NA	NA	Control
14	Branch 1	M	5	0.00	NA	NA	Normal	Normal	NA	NA	NA	NA	NA	NA	NA	NA	NA	NA	NA	NA	NA	NA	NA	NA	NA	NA	Control
15	Branch 1	M	20	0.00	NA	NA	Normal	Normal	20	79.5	52	119.5	54	93.5	NA	NA	NA	NA	NA	NA	20	38.13	38	46.5	34.5	33.5	DOA
16	Branch 1	F	5	0.10	13	0.70	Normal	Normal	7	73	37	115	51.5	89.5	16	59.5	24	96.5	46	71	16	14.50	12	17	14	15	DOA
17	Branch 1	F	8	0.00	15	0.00	Normal	Normal	4	103.5	82	118	71	144.5	12	100.5	73	112.5	73	142.5	12	52.63	47	53	56.5	54	DOA
18	Branch 1	F	4	0.22	18	0.49	Normal	B/Y Moderate	7	83.5	51.5	121.5	55	106.5	17	73.5	42.5	102.5	47.5	101.5	17	22.13	23	24	18.5	23	DOA
19	Branch 1	F	9	0.00	NA	NA	B/Y Moderate	Normal	9	108.5	77	144.5	81.5	132	NA	NA	NA	NA	NA	NA	NA	NA	NA	NA	NA	NA	Control
20	Branch 1	M	7	0.26	13	0.30	NA	NA	7	107	58.5	138.5	91	139	12	102	51	137.5	95	125.5	12	31.13	28.5	41	24.5	28.5	DOA
21	Branch 1	F	9	0.13	22	0.40	Normal	Normal	15	75	34	96.5	85	84	20	72.5	28	93	95	75.5	20	25.25	21	30	25	25	DOA
22	Branch 1	F	10	1.00	NA	NA	B/Y Moderate	B/Y Moderate	10	53	28.5	85	42	56	NA	NA	NA	NA	NA	NA	NA	NA	NA	NA	NA	NA	DOA
23	Branch 1	F	8	0.30	13	0.52	Normal	Normal	13	59	24.5	89.5	51.5	70.5	NA	NA	NA	NA	NA	NA	NA	NA	NA	NA	NA	NA	DOA
24	Branch 1	M	10	0.00	NA	NA	Normal	Normal	10	87.5	59.5	105.5	65.5	119.5	NA	NA	NA	NA	NA	NA	10	41.75	36.5	43.5	42	45	DOA
25	Branch 2	M	49	0.70	54	0.70	B/Y Moderate	B/Y Moderate	49	66	26	99.5	59.5	79	NA	NA	NA	NA	NA	NA	NA	NA	NA	NA	NA	NA	DOA
26	Branch 3	M	19	0.61	NA	NA	B/Y Moderate	B/Y Severe	NA	NA	NA	NA	NA	NA	NA	NA	NA	NA	NA	NA	NA	NA	NA	NA	NA	NA	DOA
27	Branch 4	M	11	0.00	19	0.46	Normal	Normal	12	97.5	43.5	136.5	78	132	19	82	30.5	123.5	69	105	NA	NA	NA	NA	NA	NA	DOA
28	Branch 4	F	12	0.43	16	0.55	Normal	Normal	12	67	33	102.5	37.5	95	NA	NA	NA	NA	NA	NA	NA	NA	NA	NA	NA	NA	DOA
29	Branch 5	F	15	0.56	26	0.60	B/Y Moderate	B/Y Moderate	26	51.5	26	92.5	39.5	50	NA	NA	NA	NA	NA	NA	NA	NA	NA	NA	NA	NA	DOA
30	Branch 6	M	38	0.52	46	0.60	B/Y Severe	B/Y Severe	46	63	19.5	95	63.5	74	NA	NA	NA	NA	NA	NA	NA	NA	NA	NA	NA	NA	DOA
31	Branch 6	M	24	0.35	NA	NA	NA	NA	NA	NA	NA	NA	NA	NA	NA	NA	NA	NA	NA	NA	NA	NA	NA	NA	NA	NA	DOA
32	Branch 6	F	42	1.61	48	1.65	Anarchic	Anarchic	42	45	32.5	58.5	42.5	46	48	40.5	18	50	43.5	51	NA	NA	NA	NA	NA	NA	DOA
33	Branch 6	M	9	0.80	15	0.90	Anarchic	B/Y Severe	9	62	26.5	93.5	51.5	76.5	15	57	26	89.5	48.5	64	NA	NA	NA	NA	NA	NA	DOA
34	Branch 6	F	15	0.46	21	0.70	Normal	Normal	14	48.1	44	78.5	25	45	21	41.5	36	71.5	11	47	NA	NA	NA	NA	NA	NA	DOA
35	Branch 7	M	11	0.45	31	0.70	Normal	Normal	23	65	27	108	61	64.5	27	62.5	24.5	103.5	60	63.75	27	13.50	13	15.5	12.5	14.5	DOA
36	Branch 7	M	17	0.13	25	0.45	B/Y Moderate	B/Y Moderate	25	69	35	102	51	88.5	NA	NA	NA	NA	NA	NA	25	23.50	21	27.5	22	23	DOA
37	Branch 8	M	53	0.69	67	1.00	Anarchic	Anarchic	67	57	24	85	51	67	NA	NA	NA	NA	NA	NA	NA	NA	NA	NA	NA	NA	DOA
38	Branch 8	F	26	0.25	28	0.35	Normal	Normal	28	67	38.5	80	56.5	93.5	NA	NA	NA	NA	NA	NA	28	19.63	18	23.5	14	23	DOA
39	Branch 9	F	56	0.52	NA	NA	NA	NA	NA	NA	NA	NA	NA	NA	NA	NA	NA	NA	NA	NA	NA	NA	NA	NA	NA	NA	DOA

BCVA : best corrected visual acuity; OD : *oculus dexter*; OS : *oculus sinister*; RNFL : retinal nerve fiber layer; GCL : ganglion cells layer; F : female; M: male; B : blue; Y : yellow; NA : not available. DOA : dominant optic atrophy.
