# Supplementary figures and images for: Phenotypic Variations in a Large Family with Dominant Optic Atrophy Related to a Novel OPA1 Deletion
Source: Ophthalmol Sci. 2026 Jun 15;6(8):101286. doi: 10.1016/j.xops.2026.101286 (PMC13382307; doi:10.1016/j.xops.2026.101286)

**RNFL and Age**

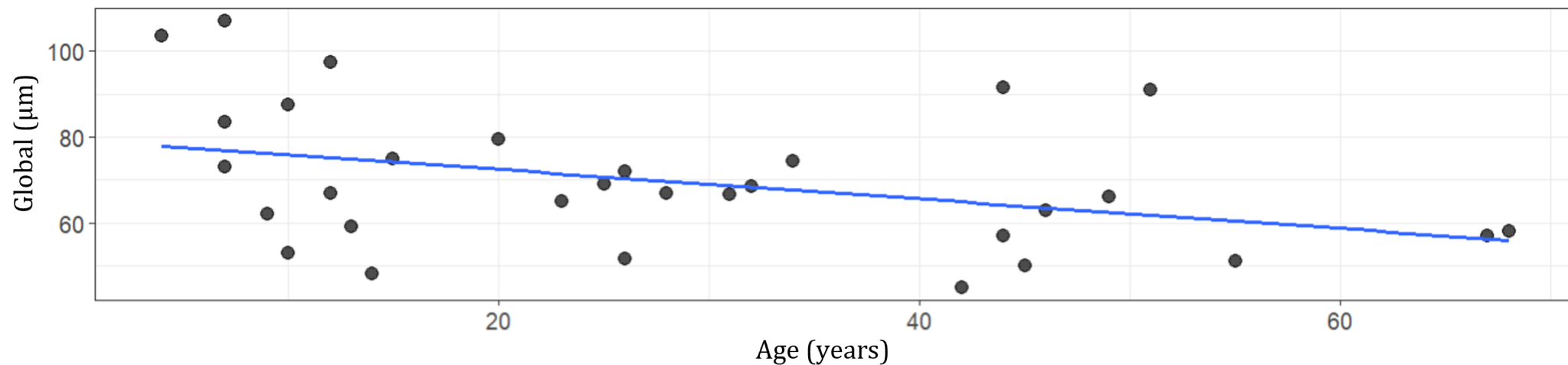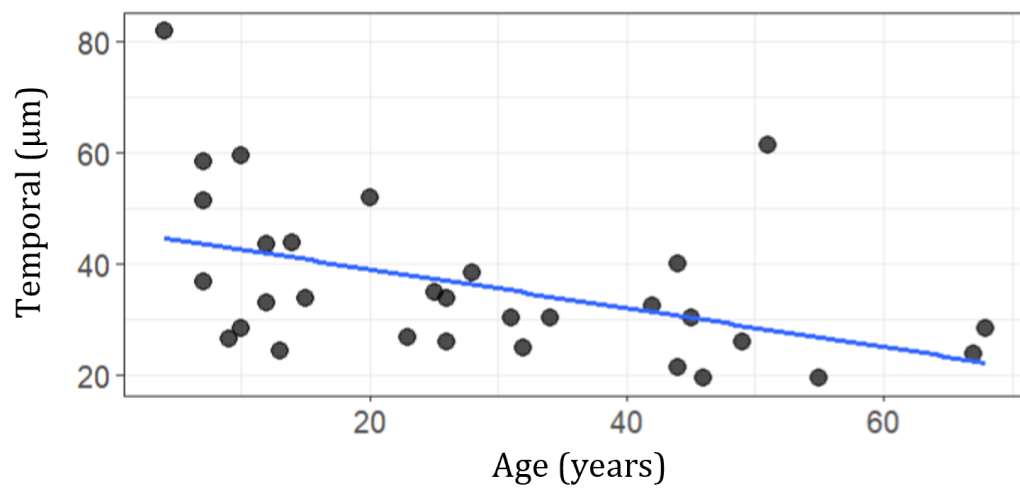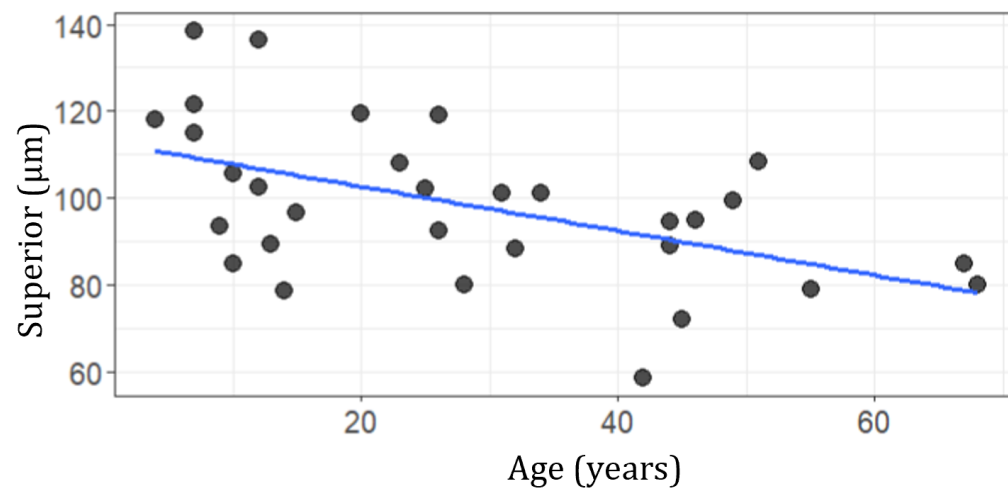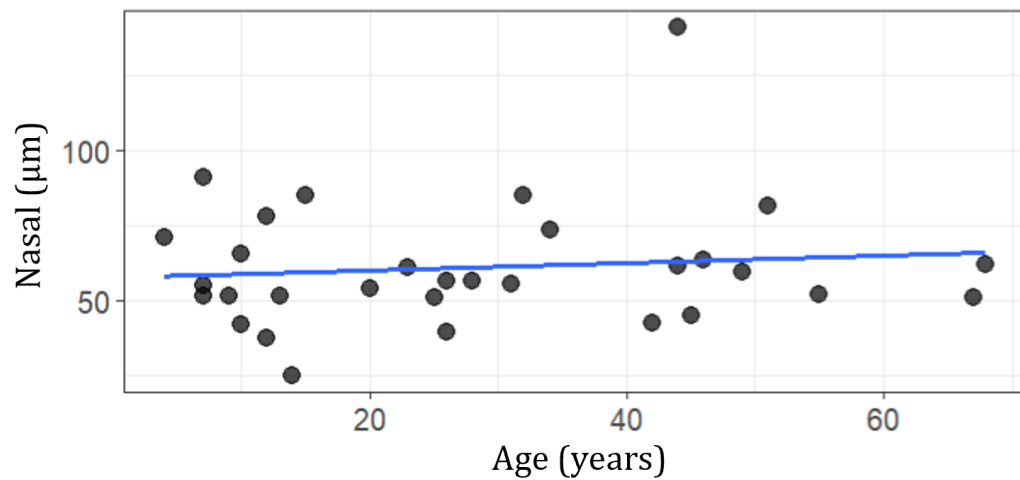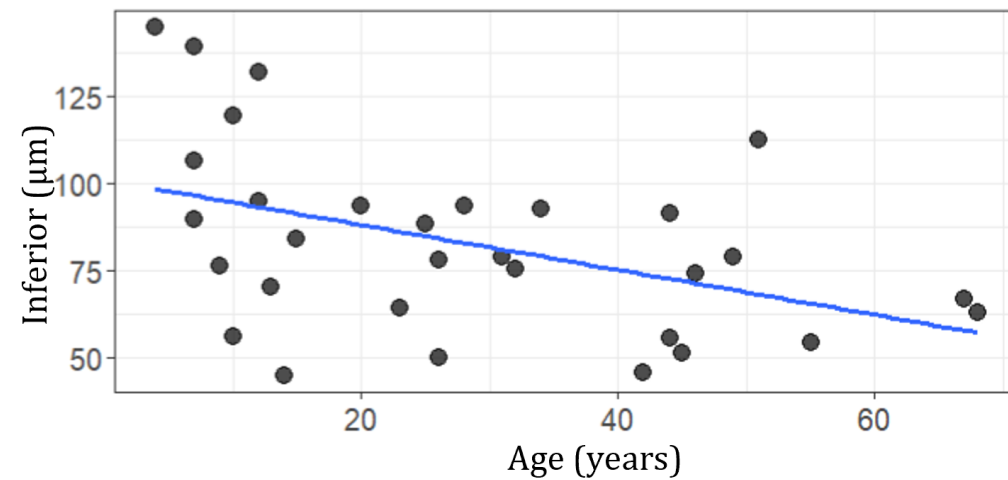

Supplement: Supplementary Table 2 [file mmc2.pdf]

## GCL and Age

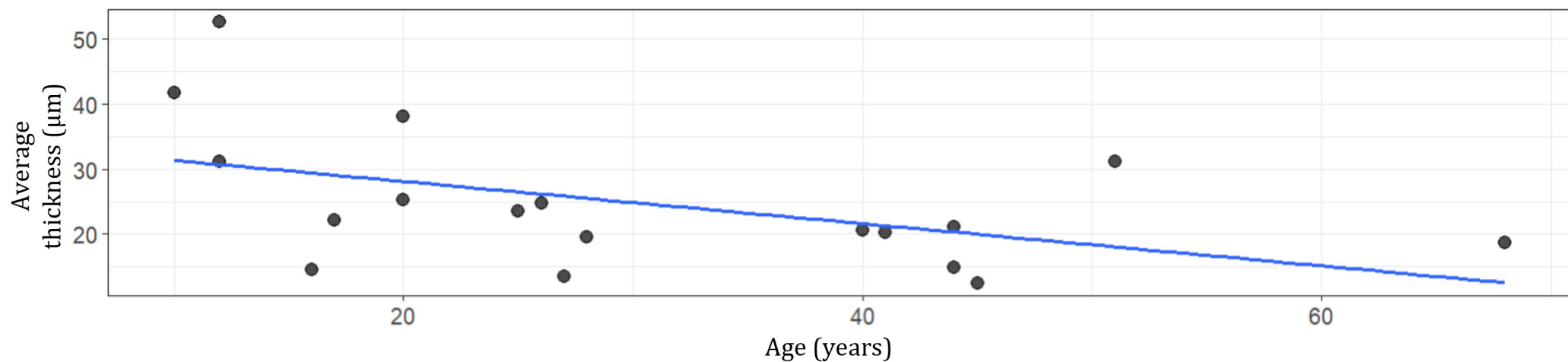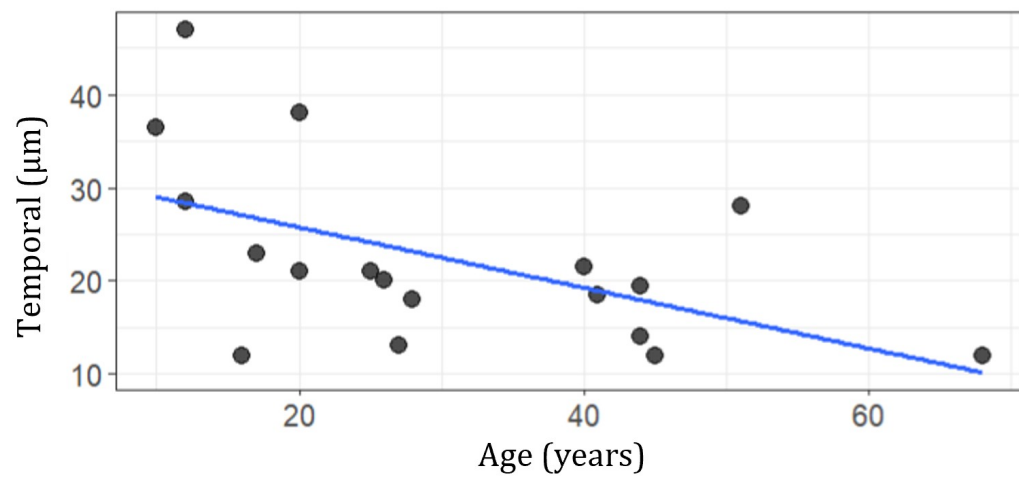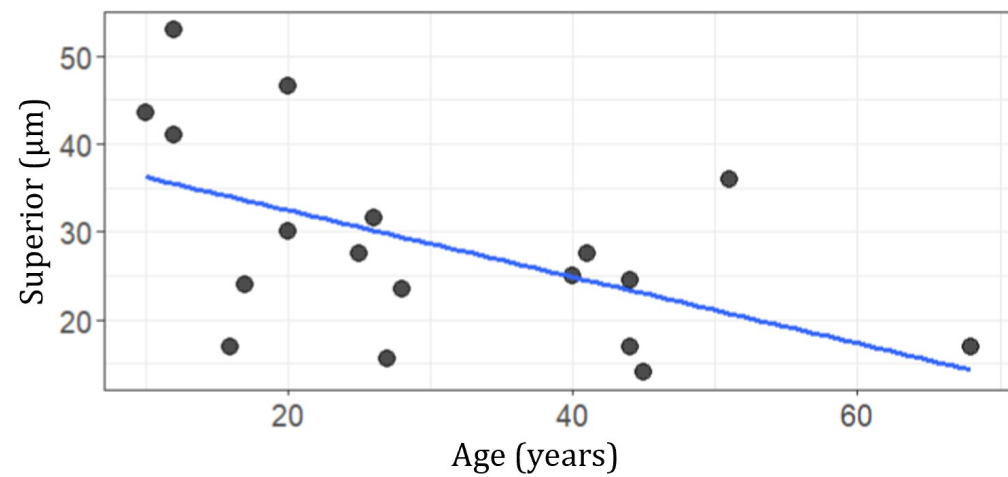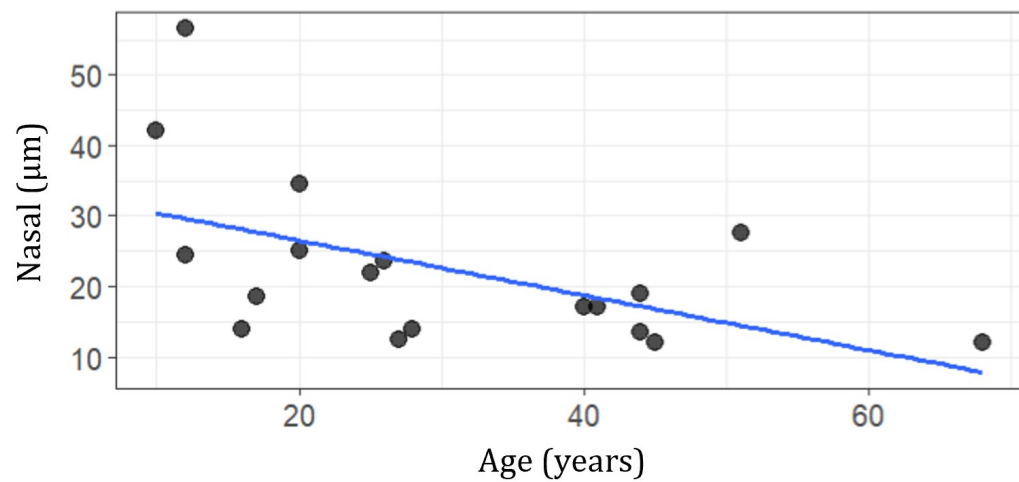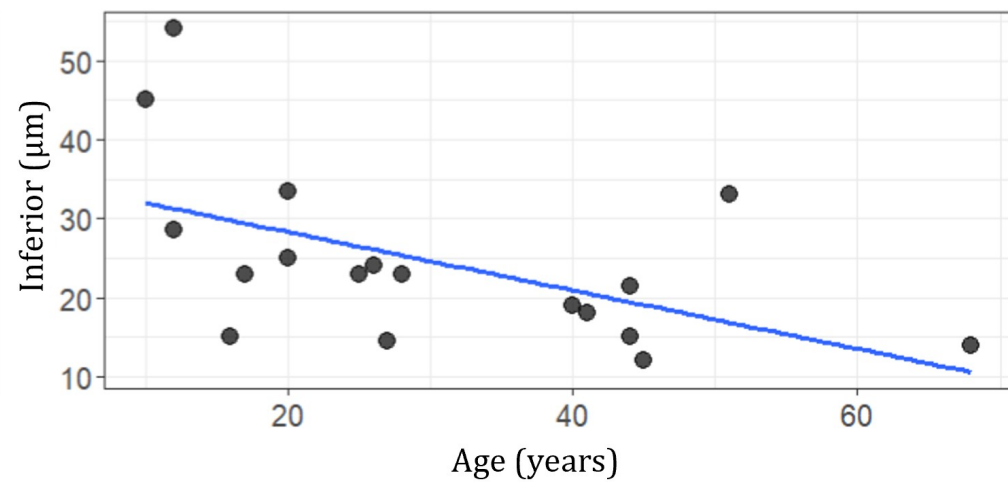

Supplement: Supplementary Table 3 [file mmc3.pdf]

## RNFL and BCVA

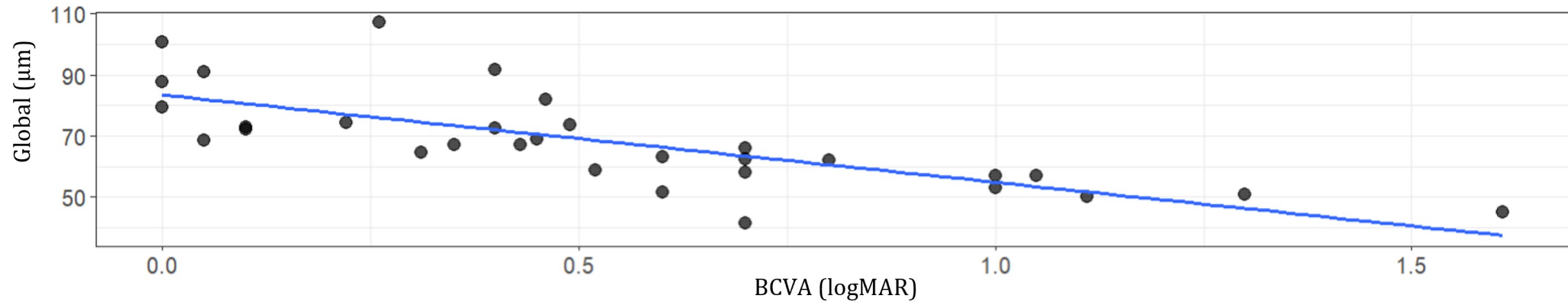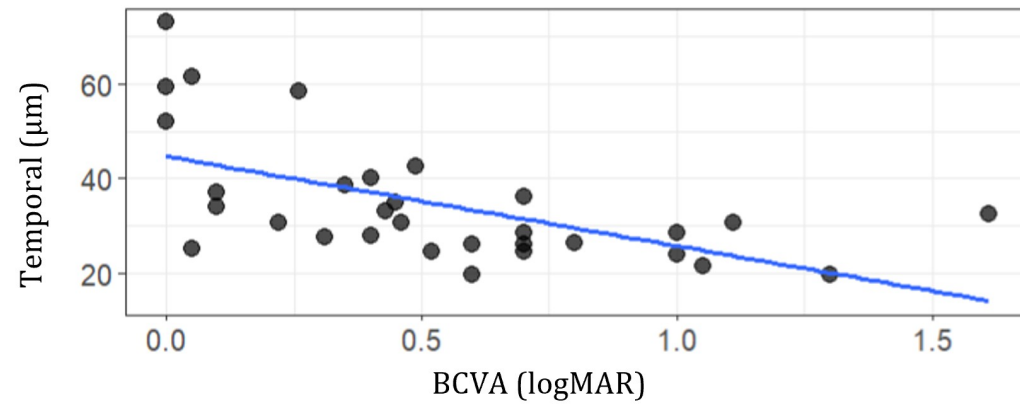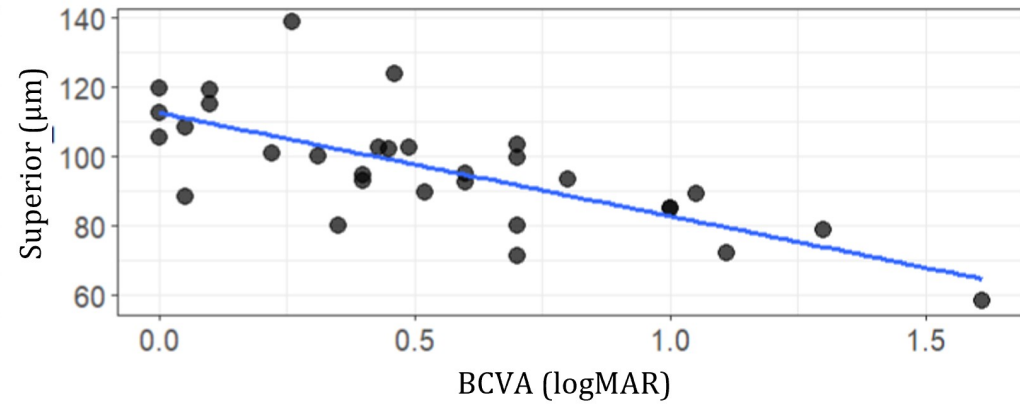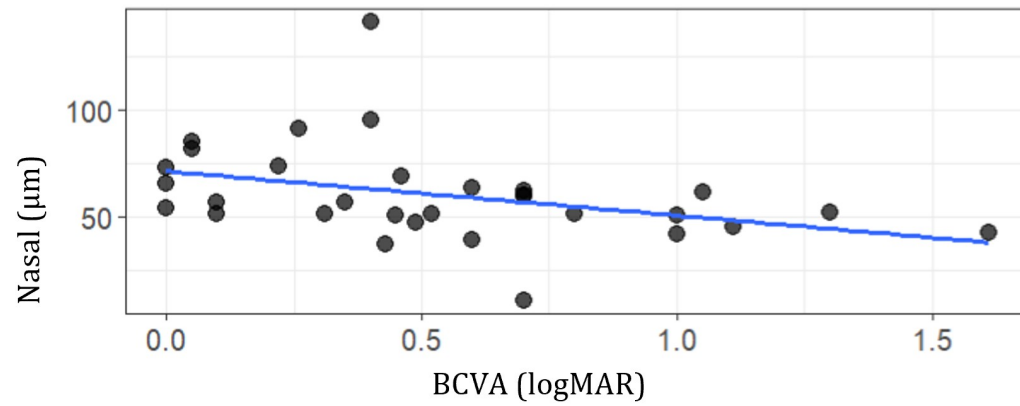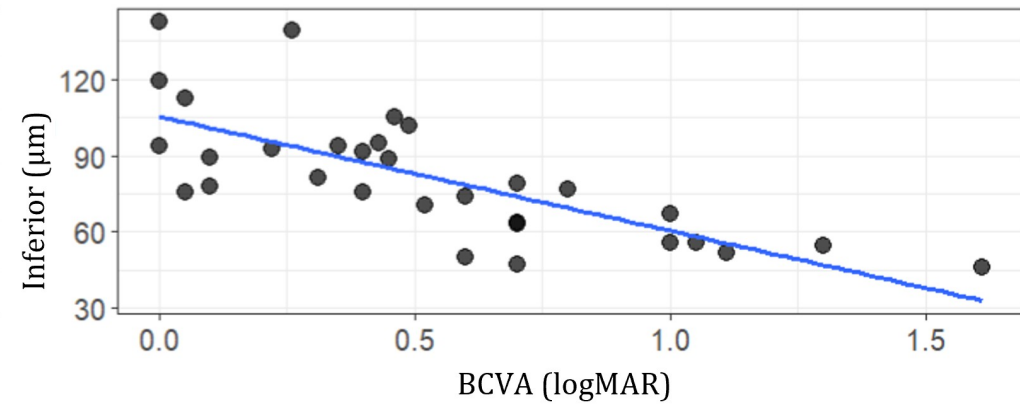

Supplement: Supplementary Table 4 [file mmc4.pdf]

## GCL and BCVA

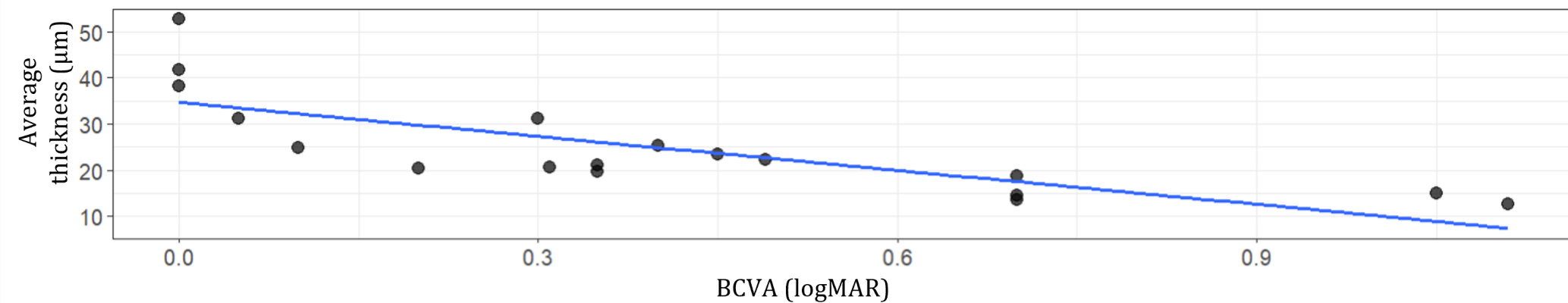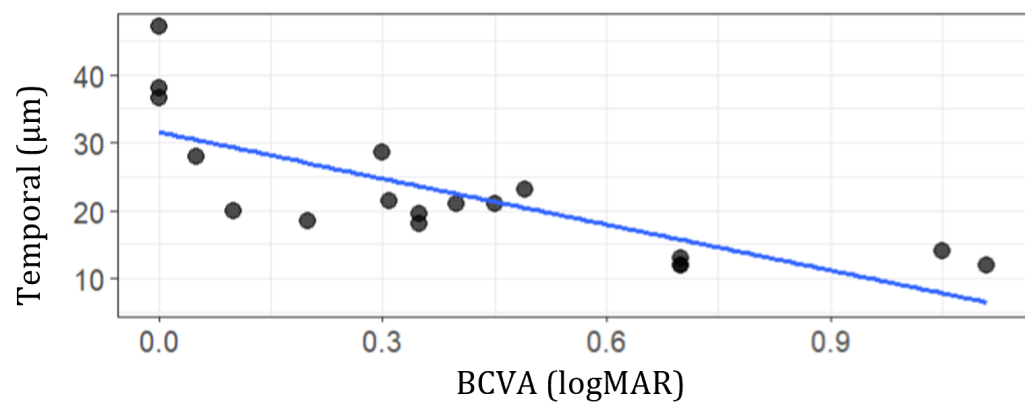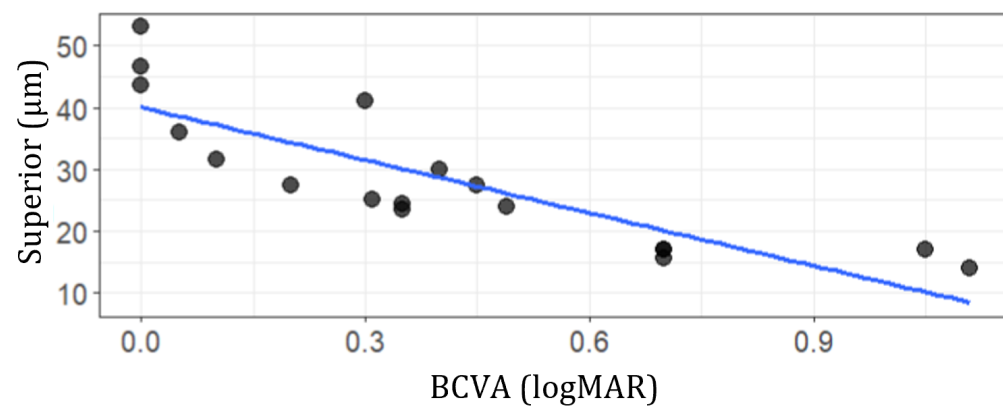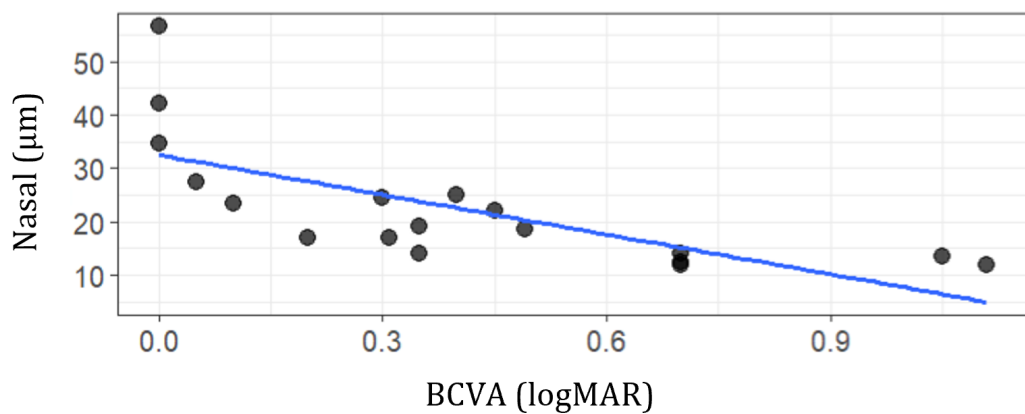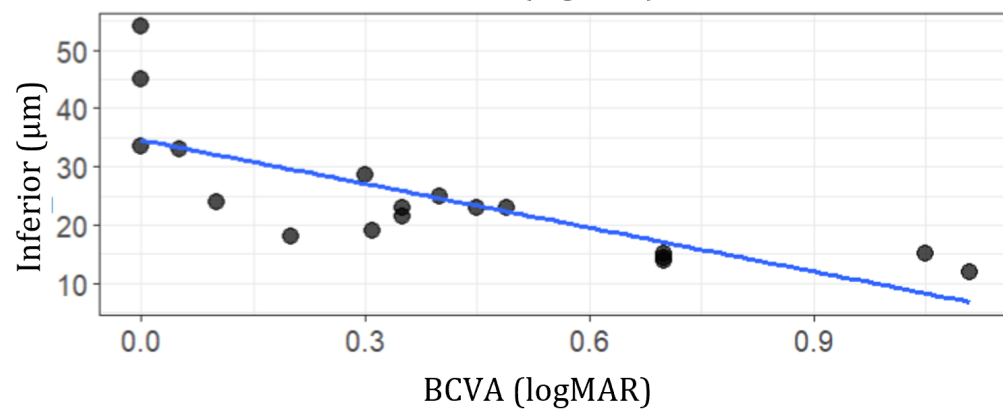

Supplement: Supplementary Table 5 [file mmc5.pdf]
